# The association of DNA-dependent protein kinase activity of peripheral blood lymphocytes with prognosis of cancer

**DOI:** 10.1038/bjc.2011.158

**Published:** 2011-05-10

**Authors:** M Someya, K-i Sakata, Y Matsumoto, R P Kamdar, M Kai, M Toyota, M Hareyama

**Affiliations:** 1 Department of Radiology, Sapporo Medical University, School of Medicine, Hokkaido, Japan; 2 Tokyo Institute of Technology, Research Laboratory for Nuclear Reactors, Tokyo, Japan; 3 Department of Biochemistry, Sapporo Medical University, School of Medicine, Hokkaido, Japan

**Keywords:** DNA-PK, peripheral blood lymphocytes, chromosomal instability, radiotherapy, prognosis, distant metastasis

## Abstract

**Background:**

Repair of various types of DNA damages is critical for genomic stability. DNA-dependent protein kinase (DNA-PK) has an important role in DNA double-strand break repair. We examined whether there may be a correlation between DNA-PK activity in peripheral blood lymphocytes (PBLs) and survival percentages in various cancer patients. We also investigated the changes of DNA-PK activity in PBLs after radiotherapy.

**Methods:**

A total of 167 of untreated cancer patients participated in this study. Peripheral blood was collected, separated, and centrifuged. DNA-PK activity was measured by DNA-pull-down assay. Chromosomal aberrations were examined by cytogenetic methods.

**Results:**

DNA-PK activity of PBLs in advanced cancer patients was significantly lower than that in early stage. The patients with lower DNA-PK activity in PBLs tended to have the lower disease-specific survivals and distant metastasis-free survivals than those with higher DNA-PK activity in advanced stages. There was also a tendency of inverse correlation between DNA-PK activity and excess fragments. The DNA-PK activity of PBLs in most patients decreased in response to radiation as the equivalent whole-body dose increased.

**Conclusion:**

Cancer patients in advanced stage, with lower DNA-PK activity of PBLs might have higher distant metastasis and exhibit poorer prognosis. Therefore, DNA-PK activity in PBLs could be used as a marker to predict the chromosomal instability and poorer prognosis.

Genomic instability is caused by constant DNA damages of various types. Of these, DNA double-strand breaks (DSBs) are believed to be one of the most serious damages induced by DNA damaging agents. DNA DSB repair has an important role in the maintenance of genomic stability (Collis *et al*, 2005).

Non-homologous end-joining (NHEJ) pathway is a prevalent mechanism for DNA DSB repair (Jeggo and Lavin, 2009). In NHEJ pathway, DSBs are directly, or after processing of the DNA ends, rejoined at an appropriate chromosomal end and DNA-dependent protein kinase (DNA-PK) has a core component of NHEJ (Lees-Miller, 1996; Jeggo, 1998). It is a serine/threonine kinase, composed of DNA-PK catalytic subunit (DNA-PKcs) and heterodimer of Ku70 and Ku86. DNA-PK binds DNA DSBs, phosphorylates, and activates DNA-binding proteins, including XRCC4, DNA ligase IV, p53, and several transcription factors. Subsequently, ligase IV reseals DNA DSBs (Jeggo, 1998).

We have previously demonstrated that DNA-PK activity in peripheral blood lymphocytes (PBLs) is associated with risk of breast and uterine cervix cancer, thereby rendering it as a marker to predict the chromosomal instability and risk of such cancers (Someya *et al*, 2006).

The PBLs have been our focus of study as they can be obtained far more easily than other human tissues. A tight correlation of DNA-PK activity between PBLs and bronchial epithelial cells (a progenitor cell for lung cancer) that were obtained by bronchoscopy, suggested that PBLs can be used as a surrogate cell type for a variety of cells (Auckley *et al*, 2001).

In this study, we examined whether there may be the relations between DNA-PK activity in PBLs and survival percentages in various cancer patients. We also investigated the changes of DNA-PK activity in PBLs, following radiotherapy as a function of the equivalent whole-body dose.

## Materials and methods

### Selection eligibility

All subjects were Japanese. In all, 167 untreated cancer patients, who were planned to receive radiotherapy at the Sapporo Medical University, were enrolled in this study. Patients with histories of other cancer, those treated with radiation or chemotherapy and those using immunosuppressive medications were excluded. Patients with breast cancer were all sporadic cases. The study was approved by the Appropriate Committees for Human Rights in Research in our hospital, and written informed consent was obtained from each subject. Individual characteristics of the patients have been summarised in Table 1. Patients with stage 0, I, or II were classified into ‘early’ and those with stage III or IV into ‘advanced’.

### Cancer treatment and estimation of radiation doses to PBLs

Breast cancer patients after partial mastectomy received postoperative irradiation to the conserved breast. Some patients were also treated with chemotherapy or hormonal therapy following radiotherapy. In uterine cervix cancer, radiotherapy consisted of external radiation and intracavitary radiation. Some patients received chemotherapy during radiotherapy. In cancer of head and neck, or oesophagus, primary radiotherapy was carried out. Some patients received chemotherapy during radiotherapy. In case of non-Hodgkin's lymphoma, primary radiotherapy was carried out. One patient received chemotherapy before radiotherapy.

In radiotherapy, the dose per fraction ranged from 1.8 to 2.0 Gy. All treatment regimens included five daily fractions per week. Several mathematical models have been developed to estimate the dose to PBLs for each treatment plan. A simple approach is to calculate the equivalent whole-body dose from the integral dose (dose × irradiated volume), divided by the body weight (Thierens *et al*, 1995). The effect of dose fractionation is not taken into account in this formula. However, it can be used in analysis of our results, as dose fractionation is constant in all cases.

### Blood collection and PBLs separation

Peripheral blood (12 ml) was collected with a sterile heparinised tube from all individuals before radiotherapy began. Blood samples were also obtained during and after radiotherapy. Lymphocytes were separated from peripheral blood cells by lymphoprep (Nycomed Pharma, Zurich, Switzerland), centrifuged at 1500 r.p.m. (300 × **g**) for 30 min at 4°C, and washed twice with phosphate-buffer saline.

### PBLs lysis, protein extraction, DNA-PK assay

Protein extraction and DNA-PK assay was carried out as described in our previous publication (Someya *et al*, 2006). Briefly, PBLs were thawed with high-salt buffer and the suspension was lysed by three rounds of freeze-thaw cycle and clarified by centrifugation at 15 000 r.p.m. (18 000 × **g**) for 7 min at 4°C. Protein concentration was assayed using a BCA protein assay kit (Thermo Fisher Scientific Inc., Barrington, IL, USA) with bovine serum albumin as the standard. The PBLs lysates were diluted to 0.25 mg ml^−1^ with high-salt buffer. The lysate was mixed with kinase assay buffer, synthetic peptide hp53-S15 (sequence: EPPLSQEAFADLWKK; synthesised in Sawady Biotechnology, Tokyo, Japan), and with or without sonicated salmon sperm DNA.

This reaction mixture was incubated at 37°C for 10 min and stopped by the addition of 30% acetic acid. The reaction mixture (40 *μ*l) was absorbed onto a P81 phosphocellulose filter (2.3 cm of diameter, Whatman, Maidstone, UK) and was washed in 15% acetic acid, and then in 99% ethanol followed by counting in a liquid scintillation counter. The net phosphorylation of hp53-S15 was calculated as phosphate incorporation in reaction with hp53-S15 minus that in reaction without hp53-S15 divided by the specific radioactivity of ATP. DNA-dependent phosphorylation of hp53-S15 is interpreted as DNA-PK activity (Lees-Miller *et al*, 1992).

### Chromosomal aberrations in PBLs

Spontaneous chromosomal aberrations in PBLs were observed by Giemsa staining from 30 patients.

Blood samples for measurements of DNA-PK activity were collected from April 2002 to August 2005. For chromosomal aberration study, we used these samples of all participants from December 2002 to March 2004. The procedure has been described previously (Someya *et al*, 2006). In all, 200 metaphase cells from each individual were analysed, and the numbers of excess fragments were counted. Chromosomal aberrations are typically subdivided into chromatid breaks and gaps *vs* chromosome breaks and gaps, triradials, quadriradials, and dicentrics. Chromosome breaks and gaps not accompanying a dicentric chromosome were defined as excess fragments in this analysis. Triradials and quadriradials were excluded from the current analysis as they were not observed.

### Western blot analysis

Cell extracts were analysed by electrophoresis on 8% polyacrylamide SDS gels, transferred to polyvinylidene difluoride membrane, and probed with a rabbit polyclonal antibody to DNA-PKcs and a mouse polyclonal antibody to Ku (Someya *et al*, 2006). Image J 1.39 (free software of National Institutes of Health, Bethesda, MD, USA) was used to analyse the results of immunoblottings.

### Statistical methods

The unpaired *t*-test was used to compare DNA-PK activity between groups. All statistical tests were two-sided. Multivariate analysis was used to clarify significant variables, which correlate with disease-specific survival or distant metastasis-free survival. The distant metastasis refers to cancer that has spread from the original (primary) tumour to distant organs or distant lymph nodes. All statistical computing was done with StatView version 4.58 (Abacus Concepts, Berkeley, CA, USA). Survival rates of the patients were measured using the Kaplan–Meier method. The overall survival was calculated from the date when the treatment started to the time of death or last follow-up. The distant metastasis-free survival was calculated from the date when the treatment started to the time of diagnosis of distant metastasis or last follow-up. The local control refers to a state of absence of any disease activity in the primary tumour. Statistical significance of survivals was compared by the log rank test.

## Results

**Figure 1:**
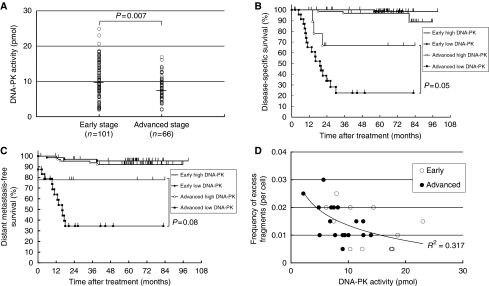
(**A**) DNA-PK activity in PBLs of patients with varied cancer types. Cancer patients were separated into two categories by stage: the early stage and the advanced stage. The horizontal bar indicates the mean value. (**B**) Disease-specific survival of cancer patients treated with radiotherapy according to stage and DNA-PK activity in PBLs. ‘Early high DNA-PK’ included 65 patients; ‘early low DNA-PK’ included 69 patients; ‘advanced high DNA-PK’ included 9 patients; ‘advanced low DNA-PK’ included 24 patients. (**C**) Distant metastasis-free survival of cancer patients treated with radiotherapy according to stage and DNA-PK activity in PBLs. (**D**) Correlation between chromosomal aberrations and DNA-PK activity of PBLs. The number of excess fragments in 200 mitotic metaphase cells was counted and expressed as the number per cell. Open circle, PBLs of patients with early stage; closed circle, that with advanced stage. The line is the regression curve.

Figure 1A compared the DNA-PK activity in PBLs of cancer patients in early and advanced stages before treatment. It was measured as 10.0±5.5 pmol (mean±s.d.) and 7.9±3.5 pmol, respectively. DNA-PK activity was found to be significantly lower in advanced cancer than in early stage (*P*=0.007). We obtained a blood sample from a normal volunteer on 11 other days from December 2002 to March 2004, and measured the DNA-PK activity of PBL to examine its intra-individual variability. It was measured as 15.0±2.8 pmol, indicating that DNA-PK activity of PBL may be relatively constant.

Figure 1B demonstrated disease-specific survival according to DNA-PK activity of PBLs when cancer patients were divided into early and advanced stages. Advanced stage patients with high DNA-PK activity demonstrated 66.7% of 5-year survival rate and 22.5% with low DNA-PK activity. Statistically, there might be a trend for better survival in high DNA-PK activity (*P*=0.05). There were no such differences observed in early stage patients. The 5-year survival rate was 96.8% with high DNA-PK activity and 98.3% with low DNA-PK activity. We further performed multiple regression analysis (Table 2). DNA-PK activity, stage, and surgery showed significant correlation with the disease-specific survival. No significant differences were found in cancer sites between breast cancer+malignant lymphomas and the other sites.

Figure 1C showed the distant metastasis-free survival in regard to DNA-PK activity of PBLs when cancer patients were divided into early and advanced stages. The 5-year distant metastasis-free survival rate in advanced stage patients was 77.8% with high DNA-PK activity and 34.5% with low DNA-PK activity. The patients with lower DNA-PK activity in PBLs tended to have the lower distant metastasis-free survivals than those with higher DNA-PK activity in advanced stages although the difference was not significant (*P*=0.08). There were no differences in early stage patients that exhibited 92.1% survival rate with high DNA-PK activity and 95.5% with low DNA-PK activity. We further performed multiple regression analysis (Table 3). In this analysis, DNA-PK activity, stage, and surgery revealed significant correlation with the distant metastasis-free survival.

The 5-year local control rate in early stage patients was 96.9% with high DNA-PK activity and 100% with low DNA-PK activity, whereas in advanced stage, it was 77.8% with high DNA-PK activity and 71.3% with low DNA-PK activity. There was no apparent correlation between local control rates with radiotherapy and DNA-PK activity of PBLs.

In this analysis of spontaneous chromosomal aberrations in PBLs at least one excess fragment was exhibited by each of the 30 patients (Figure 1D). There was a tendency of inverse correlation between DNA-PK activity and excess fragments although this correlation was not statistically significant. The frequency of excess fragment increased as DNA-PK activity decreased in PBLs. The PBLs of patients in advanced stages had the lower DNA-PK activity and the higher frequency of excess fragments than those in early stages.

**Figure 2:**
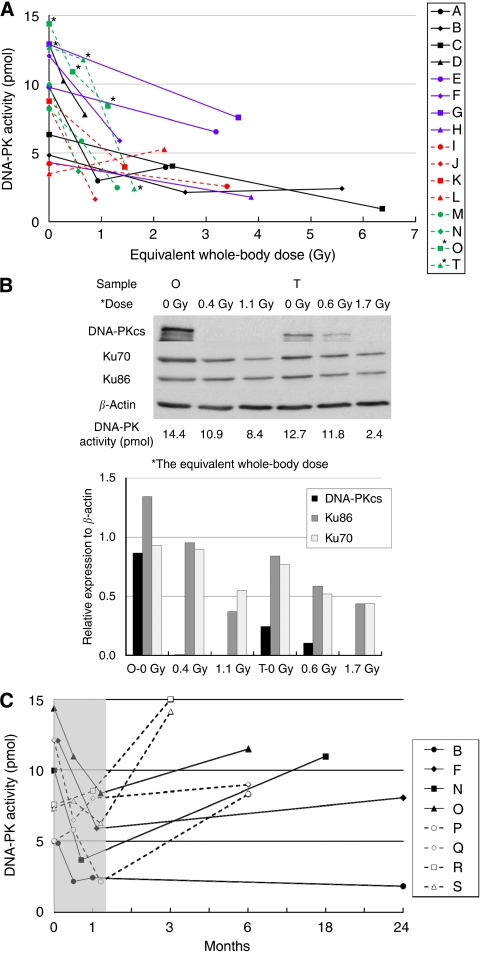
(**A**) Changes of DNA-PK activity of PBLs after radiation according to the equivalent whole-body dose. Each line indicated each patient. Each point on each line indicated the sample that was obtained at a given equivalent whole-body dose. (**B**) Alterations of DNA-PK activity and protein levels of DNA-PK genes (DNA-PKcs, Ku70, and Ku86) in PBLs of two patients (O and T in **A**) treated with radiotherapy. (**C**) Recovery of DNA-PK activity of PBLs after radiotherapy. Zero month in time means the DNA-PK activity before radiotherapy. The shaded zone in time indicates periods of radiotherapy.

Figure 2A demonstrated the alterations of DNA-PK activity of PBLs as a result of radiation in respect to the equivalent whole-body dose.

Each line indicates each patient and each marked point indicates the sample that was obtained at a given equivalent whole-body dose. The DNA-PK activity of PBLs in most patients decreased as the equivalent whole-body dose increased.

Figure 2B demonstrated changes of DNA-PK activity and protein levels of the components of DNA-PK genes (DNA-PKcs, Ku70, and Ku86) in PBLs of two patients (O and T in Figure 2A) treated with radiotherapy. DNA-PK activity and protein levels of DNA-PKcs, Ku70, and Ku86 decreased as equivalent whole-body doses increased.

Figure 2C showed the recovery of DNA-PK activity of PBLs after radiotherapy. Zero month in time denotes the DNA-PK activity measured before radiotherapy. The shaded zone in time indicates periods of radiotherapy. DNA-PK activity of PBLs in most patients was recovered after radiotherapy. However, the DNA-PK activity of PBLs of two patients (patients B and F) did not recover at 23 months after radiotherapy.

## Discussion

We have previously illustrated that DNA-PK activity in PBLs varied by a factor of 10 among the individual subjects, but the difference in DNA-PK activity in PBLs was not explained by aging or smoking history. DNA-PK activity can be influenced by the abundance of DNA-PKcs, Ku86, and Ku70 (Someya *et al*, 2006). As it is unyielding to evaluate the DNA-PK activity of infinitesimal of tumour biopsy tissues specimens, we used PBLs for the purpose. Auckley *et al* (2001) showed a tight correlation between DNA-PK activity in PBLs and bronchial epithelial cells (a progenitor cell for lung cancer) that were obtained by bronchoscopy, suggesting that PBLs can be used as a substitute cell type.

Aggressive cancer phenotypes are a manifestation of many different genetic alterations that promote rapid proliferation and metastasis (Mironchik *et al*, 2005). Genomic instability promotes a wide range of mutations, including chromosomal deletions, gene amplifications, translocations, and polyploidy. It also prompts the loss or activation of a number of critical genes, such as those involved in cell proliferation, differentiation, and apoptosis (Nishizaki *et al*, 1997; Maser and DePinho, 2002; Chin *et al*, 2004; Hezel *et al*, 2006). The repair of DSBs, the most potent lesions, is vital for stable genome maintenance. Of these, DSBs are believed to be one of the most lethal damages induced by DNA damaging agents (Collis *et al*, 2005). Unrepaired DNA ends might contribute to the development of chromosomal translocations by acting as transposable elements (Hall, 2006a). Figure 1A demonstrated that DNA-PK activities of PBLs in advanced cancer patients were significantly lower than those in early cancer. Reduced DNA-PK activity can profoundly affect the ability to repair DNA DSB, resulting in the perpetuation of chromosome damage. Our results substantiate that the lower DNA-PK activity is related with chromosomal instability (Figure 1D). This may explain as to why DNA-PK activities of PBLs in advanced cancer patients were significantly lower than those in early stage.

We found that the patients with lower DNA-PK activity in PBLs tended to have the lower survivals and higher rate of distant metastasis than those with higher DNA-PK activity in advanced stages, although the difference was not significant (Figures 1B and C). This higher tendency of distant metastasis was the key factor of their lower survival because there were no differences in local control rates between lower and higher DNA activity. Genetic instability related with low DNA-PK activity may cause higher frequency of distant metastasis. These results indicated that low DNA-PK activity in PBLs might be associated with aggressiveness of cancer phenotypes such as advanced stages and higher tendency of distant metastasis. As a result, DNA-PK activity in PBLs could function as a marker to predict the chromosomal instability and poorer prognosis to select individualised treatment for cancer.

There are several reports about expression of DNA-PK in varied tumour tissues and prognosis of patients. Lee *et al* (2005, 2007) reported that negative expression of DNA-PKcs in surgical specimens was significantly associated with tumour progression and poor patient survival rate in gastric cancers. Another report stated that DNA-PK expression of tumour tissues is negatively correlated with lymphatic metastasis, and the survival of patients with colorectal cancer. They argued that Ku70 expression might be a potential indicator for the preoperative evaluation, and prognosis in colorectal cancer (Chen *et al*, 2008). These reports lead to support our results obtained with PBLs if DNA-PK activity of PBLs represents that of other kinds of cells.

Direct correlation between local control rates with radiotherapy and DNA-PK activity of PBLs was not evident. Studies with cell lines of rodent and human origin have demonstrated that the absence of DNA-PK results in an exquisitely radiosensitive phenotype and in a reduced ability to repair DNA (Jeggo, 1998). However, dissimilar observations were made regarding whether disparity in the levels of DNA-PK activity reflect the discrepancies in inherent radiosensitivity of cell lines derived from human tumours (Allaunis-Turner *et al*, 1995; Zhao *et al*, 2000). In addition, it is unclear whether inherent radiosensitivity testing of tumour specimens is predictive of the treatment response (Allaunis-Turner *et al*, 1992; Taghian *et al*, 1993).

The DNA-PK activity along with the expressions of Ku70, Ku86, and DNA-PKcs in PBLs of most patients decreased as the equivalent whole-body dose increased (Figures 2A and B). Especially the expression of DNA-PKcs markedly decreased. The mechanism of transcriptional regulation of DNA-PK is not well known. Therefore, it is also unknown whether reduced expression of DNA-PKcs is compensated by increased expression of Ku 70 or Ku86. However, compensation for DNA-PKcs by Ku proteins may not be likely, as the role of DNA-PKcs is different from that of Ku70 and Ku86 in DNA DSB repair (Lees-Miller, 1996; Jeggo, 1998).

Decreased DNA-PK activity of PBLs after radiotherapy was recovered within several months in most of the patients. However, in two patients, it did not recover even at 23 months after radiotherapy (Figure 2C), which could be because of the protracted suppression of expression of DNA-PKcs, Ku70, or Ku86. This result suggests that DNA-PK activity of normal tissues may not recover for long periods after exposure to irradiation. Normal cells with protracted lowered DNA-PK activity after radiation exposure may be susceptible to radiation-induced cancer. It has been previously demonstrated by us that individuals with low DNA-PK activity in PBLs had increased susceptibility to cancer, such as breast and uterine cervix cancer (Someya *et al*, 2006). Intensity-modulated radiation therapy (IMRT) has been recently prevailed in clinical radiotherapy. A bigger volume of normal tissue can be exposed to lower radiation doses with IMRT as compared with other external radiation techniques. This is because IMRT exercises more monitor units and, therefore, irradiates larger equivalent whole-body doses (Hall, 2006b). Our results might indicate the importance of low dose irradiation to normal tissues in radiotherapy although its clinical meaning is not elucidated.

In summary, cancer patients with lower DNA-PK activity of PBLs tended to have the higher distant metastasis and the poorer prognosis, in advanced stage. DNA-PK activity in PBLs may thus be used as a marker to possibly predict the chromosomal instability and poorer prognosis in advanced stages patients only.

## Figures and Tables

**Table 1:** Summary of the characteristics of the patients

Age (years)	55.4 (30–88)
	
*Gender*	
Male	19
Female	148
	
*Cancer site*	
Breast	115
Uterine cervix	23
Head and neck	19
Esophagus	6
Non-Hodgkin's lymphoma	4
	
*Stage*	
Early (0–II)	134
Advanced (III–IV)	33
	
*Surgery*	
Yes	129
No	38
	
Radiation alone	34
Chemotherapy	51
Endocrinetherapy	82

**Table 2:** Multivariate analysis of prognostic factors for disease-specific survival

**Variables**	**Hazard ratio**	**95% CI**	***P*-value**	**Better prognosis**
Age	0.996	0.980–1.012	0.615	
				
*Surgery*				
Yes	0.382	0.159–0.919	0.032	Surgery
No	1			
				
*Cancer site*				
Breast+lymphoma	1			
Others^a^	2.055	0.882–4.786	0.095	Breast+lymphoma
				
*Stage*				
Early	0.173	0.064–0.467	0.001	Early stage
Advanced	1			
				
*Chemoendocrinetherapy*				
Yes	0.949	0.557–1.617	0.848	
No	1			
				
DNA-PK activity	0.934	0.900–0.968	0.001	Higher DNA-PK activity

Abbreviations: CI=confidence interval; DNA-PK=DNA-dependent protein kinase.

a Uterine cervix, esophagus, and head and neck.

**Table 3:** Multivariate analysis of prognostic factors for distant metastasis-free survival

**Variables**	**Hazard ratio**	**95% CI**	***P*-value**	**Better prognosis**
Age	0.992	0.976–1.009	0.363	
				
*Surgery*				
Yes	0.415	0.182–0.945	0.036	Surgery
No	1			
				
*Cancer site*				
Breast+lymphoma	1			
Others^a^	1.904	0.827–4.387	0.130	Breast+lymphoma
				
*Stage*				
Early	0.140	0.054–0.364	0.001	Early stage
Advanced	1			
				
*Chemoendocrinetherapy*				
Yes	0.918	0.542–1.554	0.749	
No	1			
DNA-PK activity	0.944	0.910–0.980	0.003	Higher DNA-PK activity

Abbreviations: CI=confidence interval; DNA-PK=DNA-dependent protein kinase.

a Uterine cervix, esophagus, and head and neck.
